# Simultaneous determination of three major bioactive saponins of *Panax notoginseng *using liquid chromatography-tandem mass spectrometry and a pharmacokinetic study

**DOI:** 10.1186/1749-8546-5-12

**Published:** 2010-03-23

**Authors:** Wei Chen, Yunjie Dang, Chunyan Zhu

**Affiliations:** 1 Institute of Medicinal Plant Development, Chinese Academy of Medical Sciences and Peking Union Medical College, Beijing 100193, China

## Abstract

**Background:**

Panax notoginseng saponins (PNS), the main active components of Radix Notoginseng, has been used for treating atherosclerosis, cerebral infarction, and cerebral ischemia. Ginsenosides Rg_1_, ginsenoside Rb_1_, and notoginsenoside R_1 _are the main contributors of biological activities, determination of these three saponins is very important for the *in vivo *evaluation of PNS. The present study aims to develop a liquid chromatography-tandem mass spectrometry (LC-MS/MS) method for the simultaneous quantification of ginsenosides Rg_1_, ginsenoside Rb_1_, and notoginsenoside R_1_. The use of this method was exemplified in pharmacokinetic study of beagle dog plasma after oral administration of PNS.

**Methods:**

Liquid chromatography-tandem mass spectrometry (LC/MS/MS) method was combined with solid-phase extraction (SPE). This setup was used to determine simultaneously the three major PNS (ginsenoside Rg_1_, ginsenoside Rb_1_, and notoginsenoside R_1_) in beagle dog plasma. Tandem mass spectrometry was performed using electrospray ionization in the positive ion mode.

**Results:**

The lower limits of quantification were 0.5 ng/mL for notoginsenoside R_1_, 0.82 ng/mL for ginsenoside Rg_1_, and 1.10 ng/mL for ginsenoside Rb_1_. The calibration curves for the three saponins were linear over the concentration ranges 2.64-264 ng/mL (r^2 ^= 0.9967, P = 0.003), 3.6-360 ng/mL (r^2 ^= 0.9941, P = 0.004), and 18.7-1870 ng/mL (r^2 ^= 0.9912, P = 0.004) for notoginsenoside R_1_, ginsenoside Rg_1_, and ginsenoside Rb_1_, respectively. Within these concentration ranges, the relative standard deviation (RSD) of intra- and interday assays for the three PNS from beagle dog plasma samples were less than 12%.

**Conclusions:**

This LC/MS/MS method in combination with SPE is useful in the pharmacokinetic study of PNS, such as the simultaneous determination of saponins in beagle dog plasma after oral administration.

## Background

**Figure 1:**
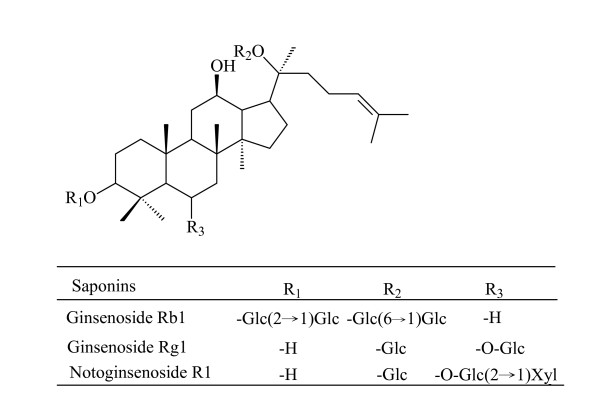
Chemical structures of ginsenoside Rb_1_, ginsenoside Rg_1_, and notoginsenoside R_1_

*Panax notoginseng *saponins (PNS), the main active components of *Radix Notoginseng *[1], are used for treating atherosclerosis [2], cerebral infarction [3], and cerebral ischemia [4]. The major bioactive saponins of *Radix Notoginseng *[5] are ginsenoside Rg_1_, ginsenoside Rb_1_, and notoginsenoside R_1 _(Figure 1). In addition, notoginsenoside R_1_, a saponin unique to *Panax notoginseng*, has anti-thrombus activity [6]. Thus, determination of these three saponins is very important for the *in vivo *monitoring and evaluation of PNS.

Determination of the limit of quantification (LOQ) of PNS in biological samples is a great challenge for *in vivo *research. The reported quantitative methods included thin-layer chromatography [7,8], high-performance liquid chromatography (HPLC) with UV detection [9,10], fluorescence detection [11], gas chromatography-mass spectrometry [12], and liquid chromatography-mass spectrometry (LC/MS) with electrospray positive [13] or negative [14] ionization. Although many methods were developed to determine the saponins of PNS in extracts, commercial products, and biological samples, the LOQ values were unsatisfactorily determined in biological samples. For example, the LOQ values of ginsenoside Rg_1 _and ginsenoside Rb_1 _were higher than 500 ng/mL in rat biological samples detected by thin-layer chromatography [7,8]; 60 and 5.0 ng/mL, respectively, in rat urine samples by HPLC-UV [15]; and 10.0 ng/mL using a 100- μl plasma sample by LC/MS with negative ionization [10]. There is no report on the determination of notoginsenoside R_1 _after oral administration of PNS or the simultaneous determination of ginsenosides Rg_1 _and Rb_1 _and notoginsenoside R_1 _in plasma after oral administration of PNS. Thus, it is necessary to develop a sensitive method to determine the saponins in PNS, especially in biological samples.

As LC-MS is useful in drug metabolism and pharmacokinetic studies on active components in Chinese medicines [16-19], we developed a LC-MS method for drug metabolism and pharmacokinetic study of ginsenoside Rg_3 _and ginsenoside Rh_2_, and our findings showed that LC-MS and MS/MS methods are sensitive, specific, and convenient for such ginsenoside studies [17-19].

Multiple reactions monitoring (MRM) mass spectrometry has special advantages for quantitative analysis of analyte: it is specific and selective technology that can avoid contaminants in the biological samples. In the present study, we aims to develop a liquid chromatography-tandem mass spectrometry (LC-MS/MS) multiple reactions monitoring method combined with solid-phase extraction preparation of samples for the simultaneous quantification of ginsenosides Rg_1_, ginsenoside Rb_1_, and notoginsenoside R_1 _and to conduct a pharmacokinetic study of beagle dog plasma after oral administration of PNS.

## Methods

### Chemicals and reagents

Acetonitrile and methanol (HPLC grade) were purchased from Fisher Scientific (NewJersey, USA). Extract-Clean C_18 _(solid-phase extraction) cartridge columns were purchased from the Alltech Company (Deerfield, USA). Ginsenosides Rg_1 _and Rb_1 _and notoginsenoside R_1 _were purchased from the National Institute for the Control of Pharmaceutical and Biological Products (Beijing, China), and PNS extract was purchased from the Institute of Medicinal Plant Development, Yunnan Branch (Yunnan Province, China). The contents of ginsenosides Rg_1 _and Rb_1 _and notoginsenoside R_1 _in the PNS extract were 37%, 36%, and 10%, respectively, according to HPLC analysis. Glipizide (internal standard) was kindly provided by Professor Jifen Guo at the Academy of Military Medical Sciences (Beijing, China).

### Instrumental analysis

The HPLC system consisted of a quaternary LC pump of Agilent 1100 series, from Agilent Technology (Waldbronn, Germany), with a degasser and an autosampler. The mobile phase consisted of 10 mM ammonium acetate solution, 0.1% n-butyl-amine,10% methanol in water (A) and methanol (B). The flow rate was kept at 0.2 mL/min for a total run time of eight minutes. The system was run with a gradient program of 50% B to 90% B in five minutes and 90% B to 50% B in three minutes. The samples were introduced into the electrospray ion source of a triple-stage quadrupole mass spectrometer Finnigan TSQ 7000 with API-2 ion source and performance kit (Thermo Electron, Dreieich, Germany). Electrospray ionization interface parameters were as follows: spray voltage: 4.0 kV; sheath gas (N_2_): 8 units; capillary heater temperature: 300°C. Multiple reactions monitoring measurements were performed at a multiplier voltage of 1.2 kV.

### Animals

Three male beagle dogs, weighing 10-12 kg, were purchased from Tongli Laboratory Animals Center (Beijing, China) for the animal experiments. Experimental animals were maintained in accordance with internationally guidelines for laboratory animal use [20], and the study was approved by the Beijing Animal Care Committee (Beijing China).

### Sample preparation

For the assay, a 0.5 mL plasma sample was loaded and drawn through by gravity on a solid-phase extraction cartridge (1 mL, packed with 100 mg of 40 μm octadecyl silica) that was preconditioned by passing through 2 mL of methanol followed by 2 mL of water before loading. Then the solid-phase cartridge was washed with 2 mL of water, 2 mL of 20% (v/v) aqueous methanol solution, and 2 mL of methanol. The final methanol elute was collected and 10 μl of internal standard was added to it. The elute was thereafter evaporated at 40°C to dryness under a stream of nitrogen. The residue was dissolved in 100 μl of 50% (v/v) aqueous methanol solution. A 20 μl sample was injected into the HPLC system for analysis.

### Method validation

#### Calibration curve and quality control sample preparation

Primary mixed stock solutions of notoginsenoside R_1_, ginsenoside Rg_1_, and ginsenoside Rb_1 _were prepared in methanol/water (1:1, v/v). Working standard solutions of the three saponins were prepared by combining the aliquots of each primary solution and diluting with methanol/water (1:1, v/v). The working solution for internal standard (0.1 μg/mL) was prepared using methanol. All stock solutions were stored at 4°C in polypropylene tubes in the dark.

Beagle dog plasma calibration standards of the three saponins were prepared by spiking standard solutions with various concentrations of drug-free beagle dog plasma. Standard solutions of six different concentrations of the three saponins were obtained: 2.64, 5.28, 13.2, 26.4, 52.8, and 264 ng/mL for notoginsenoside R_1_; 3.60, 7.20, 18.0, 36.0, 72.0, and 360 ng/mL for ginsenoside Rg_1_; and 18.7, 37.4, 93.5, 187, 374, and 1870 ng/mL for ginsenoside Rb_1_. Three calibration curves were established by determining the peak area ratio (analyte/internal standard) versus the saponin concentrations in samples processed as described in *Sample preparation*.

Quality control samples were prepared in bulk by adding 50 μl of the appropriate working standard solutions to blank beagle dog plasma. The quality control samples at 2.8, 14, and 140 ng/mL for notoginsenoside R_1_; 3.8, 19, and 190 ng/mL for ginsenoside Rg_1_; and 18, 180, and 900 ng/mL for ginsenoside Rb_1 _were stored at -20°C until analysis.

#### Accuracy and precision

Batches of quality control samples were analyzed on four different days to validate the method. In each batch, quality control samples were assayed in sets of six replicates to evaluate the accuracy and intra- and interday precision. The accuracy and relative standard deviation (RSD) were used to evaluate the method.

#### Recovery and sensitivity

The relative recovery of this method was determined by comparing the concentration calculated from the calibration curve to the known concentration. Recovery was evaluated with four replicates, and samples were prepared as the quality control samples.

The LOQ values were measured using a series of diluted standard plasma samples. For the concentration to be accepted as the LOQ, the signal-to-noise ratio had to be greater than or equal to five and the percent deviation for the analyte concentration and the relative standard deviation had to be within 20%.

#### Pharmacokinetic study

PNS extract was administered orally at a dose of 90 mg/kg to beagle dogsthat had been fasting but had free access to water for 18 hours prior to the experiment. Each blood sample (3 mL) was transferred to a heparinized glass tube at 0 hr, 0.5 hr, 1 hr, 2 hr, 3 hr, 4 hr, 6 hr, 8 hr, 10 hr, 12 hr, 16 hr, 24 hr, 36 hr, 48 hr and 72 hr after administration and immediately centrifuged at 1500 × *g *for 15 minutes at 8-10°C. The plasma was then transferred to another glass tube and stored at -20°C until analysis.

## Results and discussion

### Sample preparation

Sugar moieties are extracted incompletely into organic solvents. Therefore, solid-phase extraction was used to remove excess ingredients in plasma and achieve good selectivity. When the concentration of methanol was below 20%, the three saponins were strongly absorbed by the C_18 _stationary phase, while the interfering compounds in plasma could be eluted under this condition. The excellent selectivity of solid-phase extraction helped to improve the LC-MS/MS analysis.

### Performance and validation of the analytical method

**Figure 2:**
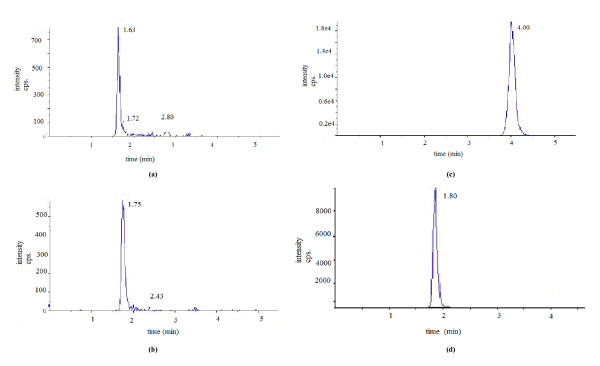
**HPLC chromatograms of (a) notoginsenoside R_1_; (b) ginsenoside Rg_1_; (c) ginsenoside Rb_1_; and (d) internal standard (glipizide)**.

The three saponins and internal standard in plasma were completely separated within six minutes without significant interference (Figure 2), demonstrating the specificity of this method. The retention times were as follows: 1.56, 1.67, 3.87, and 1.80 minutes for notoginsenoside R_1_, ginsenoside Rg_1_, ginsenoside Rb_1_, and internal standard, respectively.

**Figure 3:**
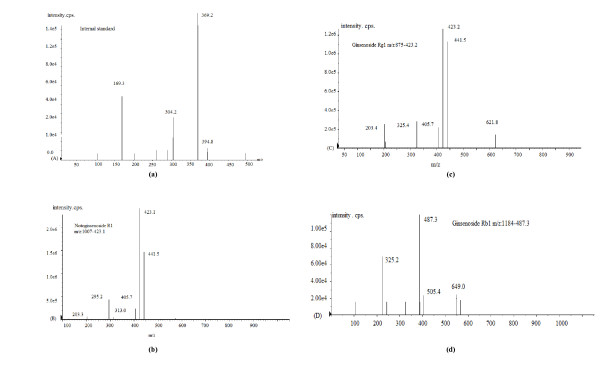
**Tandem mass spectra (daughter ion scans) of (a) internal standard (glipizide); (b) notoginsenoside R_1 _parent ion; (c) ginsenoside Rg_1_; and (d) ginsenoside Rb_1_**.

MS/MS transitions monitored in the positive ion mode were m/z 1007 → m/z 423 for notoginsenoside R_1 _(MW 932), m/z 875 → m/z 423 for ginsenoside Rg_1 _(MW 800), m/z 1183 → m/z 487 for ginsenoside Rb_1 _(MW 1108), and m/z 494 → m/z 369.1 for internal standard. MS/MS spectra (daughter ion scans) of the three saponins and internal standard are shown in Figure 3.

In this method, standard curves are linear over the range of 2.64-264 ng/mL for notoginsenoside R_1_, 3.60-360 ng/mL for ginsenoside Rg_1_, and 18.7-1870 ng/mL for ginsenoside Rb_1_, with all correlation coefficients larger than 0.99. The LOQ values are 0.5 mL for notoginsenoside R_1_, 0.82 ng/mL for ginsenoside Rg_1_, and 1.10 ng/mL for ginsenoside Rb_1_. The results of the analysis are shown in Table 1.

**Table 1:** Calibration curves of the three *Panax notoginseng *saponins in beagle dog plasma

Analyte	Standard curves (linear)	*P*	*r*^2^	Test range (ng/mL)	Limit of quantification (ng/mL)
NotoginsenosideR_1_	*Y *= 0.0011*X*+0.0004	0.003	0.9967	2.64--264	0.50
Ginsenoside Rg_1_	*Y *= 0.0009*X*+0.0007	0.004	0.9941	3.60--360	0.82
Ginsenoside Rb_1_	*Y *= 0.0016*X*+ 0.0283	0.004	0.9912	18.7--1870	1.10

Note: *Y*, peak area ratio (analyte/internal standard); *X*, concentration of analyte in plasma (ng/mL)

The results of the assay of precision and accuracy are shown in Table 2. The relative standard deviation of the method was less than 12.0%. Recovery test results are shown in Table 3.

**Table 2:** Intra- and interday variability for the assay of the three *Panax notoginseng *saponins in beagle dog plasma (*n *= 6)

**Spiked conc**. (ng/mL)	Intraday			Interday		
		
	Measured conc. (ng/mL)	RSD (%)^a^	Accuracy (%)^b^	Measured conc. (ng/mL)	RSD (%)	Accuracy (%)
Notoginsenoside R_1_						
2.8	2.8	1.27	100.0	2.68	1.49	95.71
14	13.9	0.76	99.29	12.89	9.62	92.1
140	148	8.22	105.7	131.67	10.11	105.7
Ginsenoside Rg_1_						
3.8	3.6	5.13	94.7	3.96	11.96	104.2
19	17.6	3.65	92.6	18.3	8.95	96.3
190	197.3	6.59	103.8	193.7	7.66	101.7
Ginsenoside Rb_1_						
18	18.1	3.07	100.6	17.6	5.36	96.11
180	165.3	2.73	91.83	179.7	5.05	99.50
900	912.67	3.90	101.3	871.3	2.38	96.87

Note: ^a^Relative standard deviation; ^b^Accuracy (%) = [1--(nominal concentration -- mean of measured concentration)/nominal concentration] × 100

**Table 3:** Recovery of the three *Panax notoginseng *saponins from beagle dog plasma (*n *= 4)

Analyte	Spiked conc. (ng/mL)	Recovery (%)^a, b^	RSD (%)^c^
Notoginsenoside R_1_	2.8	96.0 ± 6.72	6.99
	14	104.0 ± 7.71	7.41
	140	102.3 ± 7.82	7.82
Ginsenoside Rg_1_	3.8	99.9 ± 6.89	6.90
	19	102.4 ± 6.73	6.57
	190	98.96 ± 8.06	8.14
Ginsenoside Rb_1_	18	102.6 ± 9.85	9.60
	180	101.1 ± 10.26	9.14
	900	105.0 ± 8.32	7.92

Note: ^a^Recovery (%) = [1 -- (spiked concentration -- measured concentration)/spiked concentration] × 100; ^b^Values are the mean ± SD (*n *= 4); ^c ^Relative standard deviation

### Pharmacokinetics study of the three saponins

The pharmacokinetic parameters are listed in Table 4. In this study, notoginsenoside R_1 _and ginsenoside Rg_1 _could hardly be detected after 24 hours, whereas ginsenoside Rb_1 _could still be detected at four days. Thus, the half-life of ginsenoside Rb_1 _is much longer than those of ginsenoside Rg_1 _and notoginsenoside R_1_.

**Table 4:** Pharmacokinetic parameters of the three saponins

Parameters	Notoginsenoside R_1_	Ginsenoside Rg_1_	Ginsenoside Rb_1_
C_max _(ng/mL)	37.57 ± 24.55	95.57 ± 69.30	1080.00 ± 186.82
T_max _(h)	2.67 ± 1.15	2.63 ± 1.15	8.67 ± 6.43
T_1/2 _(h)	3.35 ± 0.55	4.55. ± 1.22	18.274 ± 2.55
AUC	107.02 ± 17.39	233.42 ± 87.69	35178.68 ± 12236.26

## Conclusions

This LC-MS/MS assay combined with solid-phase extraction was simple, rapid, highly sensitive, and precise for the determination of notoginsenoside R_1_, ginsenoside Rg_1_, and ginsenoside Rb_1 _in beagle dog plasma samples after oral administration of PNS at 90 mg/kg. Although thin-layer chromatography and HPLC-UV methods had been used to study the pharmacokinetics of saponins in PNS [5,6], the specific and more sensitive LC-MS/MS method allows for the simultaneous determination of the three saponins and the quantification of PNS in biological samples, making this as a promising method for the *in vivo *study of PNS.

## Abbreviations

PNS: *Panax notoginseng *saponins; LC-MS/MS: liquid chromatography-tandem mass spectrometry; HPLC: high-performance liquid chromatography; SPE: solid-phase extraction; MRM: Multiple reaction monitoring; IS: internal standard; QC: quality control; LOQ: limit of quantification; RSD: Relative standard deviation.

## Competing interests

The authors declare that they have no competing interests.

## Authors' contributions

WC designed the study, conducted the animal experiments and performed the statistical analysis. YJD drafted the manuscript. CYZ conceived the study and participated in its design. All authors read and approved the final manuscript.
